# Whole-tissue imaging reveals intrastrain diversity shapes the spatial organization of *Pseudomonas aeruginosa* in a murine infection model

**DOI:** 10.1128/msphere.00657-25

**Published:** 2025-12-16

**Authors:** H. L. Fraser, D. A. Moustafa, J. B. Goldberg, S. Azimi

**Affiliations:** 1 Biology Department, College of Arts and Sciences, Georgia State University 1373, Atlanta, Georgia, USA; 2 Department of Pediatrics, Division of Pulmonary, Asthma, Cystic Fibrosis, and Sleep, Emory University School of Medicine 12239, Atlanta, Georgia, USA; 3 Emory+Children's Cystic Fibrosis Center 200748 , Atlanta, Georgia, USA; University of Galway, Galway, Ireland

**Keywords:** *Pseudomonas aeruginosa*, intrastrain genetic diversity, cystic fibrosis, HCR, aggregate, whole-tissue imaging

## Abstract

Intrastrain genetic and phenotypic heterogeneity of *Pseudomonas aeruginosa* is a hallmark of chronic lung infections in individuals with cystic fibrosis (CF) and chronic obstructive pulmonary disease (COPD). Although the coexistence of multiple *P. aeruginosa* lineages within a single host is well documented, the impact of this heterogeneity on infection microbiogeography remains poorly understood. We previously showed that loss of the lipopolysaccharide (LPS) O-specific antigen (OSA) alters *P. aeruginosa* aggregate assembly. Since OSA-deficient variants are common in chronic pulmonary infections and associated with increased pathogenesis and immune evasion, we investigated whether intrastrain OSA diversity shapes infection microbiogeography. We constructed mixed populations containing equal ratios of OSA-deficient variants and wild-type (WT) cells and examined aggregate assembly and population structures in a synthetic CF sputum model (SCFM2). To assess OSA heterogeneity *in vivo*, we used a murine pneumonia model combined with hybridization chain reaction (HCR) RNA-FISH and whole-tissue clearing to visualize spatial organization in the airways. In SCFM2, OSA-deficient variants increased total population size, reduced WT aggregate size, and altered spatial organization. We employed 2-plex HCR RNA-FISH to distinguish WT and OSA-deficient variants in murine lungs. Interestingly, in contrast to *in vitro* conditions, OSA-deficient cells led to significantly larger WT aggregates in the airways. These findings highlight the role of intrastrain genetic heterogeneity in shaping infection microbiogeography and provide a framework for understanding how population dynamics influence microbial physiology and host-pathogen interactions at the micron scale.

**IMPORTANCE:**

Intrastrain genetic and phenotypic diversity within *Pseudomonas aeruginosa* populations is common in chronic pulmonary infections. While this intrastrain heterogeneity is a hallmark of chronic infection, its consequences for the spatial organization of *P. aeruginosa* within the airways remain unclear. Here, we demonstrate that the loss of O-specific antigen in a subpopulation of *P. aeruginosa* significantly alters the spatial architecture of *P. aeruginosa*, without changing the total population size or composition. Using a combination of tissue clearing and hybridization chain reaction RNA-FISH in a murine lung infection model, we mapped the localization of genetically distinct *P. aeruginosa* variants in mixed populations *in vivo*. These findings reveal that genetic diversification within a strain can reshape the infection landscape at the micron scale, highlighting the overlooked role of intrastrain dynamics in shaping the microbiogeography of infections and influencing host-pathogen interactions.

## OBSERVATION

Chronic infections are typically polymicrobial, with bacteria living in surface-attached biofilms or as suspended aggregates of 10–10⁴ cells, often exhibiting defined spatial patterning (1–3). This spatial patterning at the micron scale (microbiogeography) is shaped by and can be impacted by changes in bacterial population dynamics, microbial community structure, and host factors. Despite the important role of microbiogeography in pathogenesis and treatment outcomes, mechanisms regulating the microbiogeography of chronic infections remain poorly understood.

In people with cystic fibrosis and chronic pulmonary obstructive disease, chronic *Pseudomonas aeruginosa* lung infection is linked with high rates of pulmonary exacerbations and decreased life expectancy (4, 5). Cystic fibrosis (CF) lung disease is characterized by increased inflammatory responses due to the dysfunction of the cystic fibrosis transmembrane conductance regulator in bronchial epithelial cells, compounded by impaired mucociliary clearance of inhaled bacteria (6, 7). Notably, airway inflammation and bronchiectasis are not uniformly distributed across the CF lungs*,* and computed tomography scans often exhibit focal obstructions in various lung regions, suggesting heterogeneity in the physiological and physicochemical properties of the airway environment (2, 8–11). This uneven distribution in airway thickening and damage suggests that differences in the microbial population residing in various parts of the lungs can influence the inflammatory responses and physiology of airway epithelial cells at the micron scale. Although within-host adaptation of *P. aeruginosa* and co-occurrence of genetically distinct variants within the CF lungs are well documented (4, 12, 13), their consequences on spatial organization and microbiogeography of airways are not fully understood. Our previous studies showed that loss-of-function mutations in the *ssg* and *wbpL* genes alter *P. aeruginosa* aggregate assembly in a synthetic *in vitro* preclinical model of CF sputum (SCFM2) (3, 14, 15). These mutations led to loss of O-specific antigen (OSA) and increased cell surface hydrophobicity. OSA-deficient variants, which are described as lipopolysaccharide (LPS)-rough, are frequently isolated alongside those with an LPS-smooth phenotype from CF sputum and associated with increased inflammation and evasion of the immune system (16–19), signifying that intrastrain phenotypic and genetic heterogeneity in OSA may contribute to *P. aeruginosa* population spatial organization in airways.

### Presence of OSA-deficient cells alters aggregate assembly of *P. aeruginosa* populations *in vitro*

**Fig 1:**
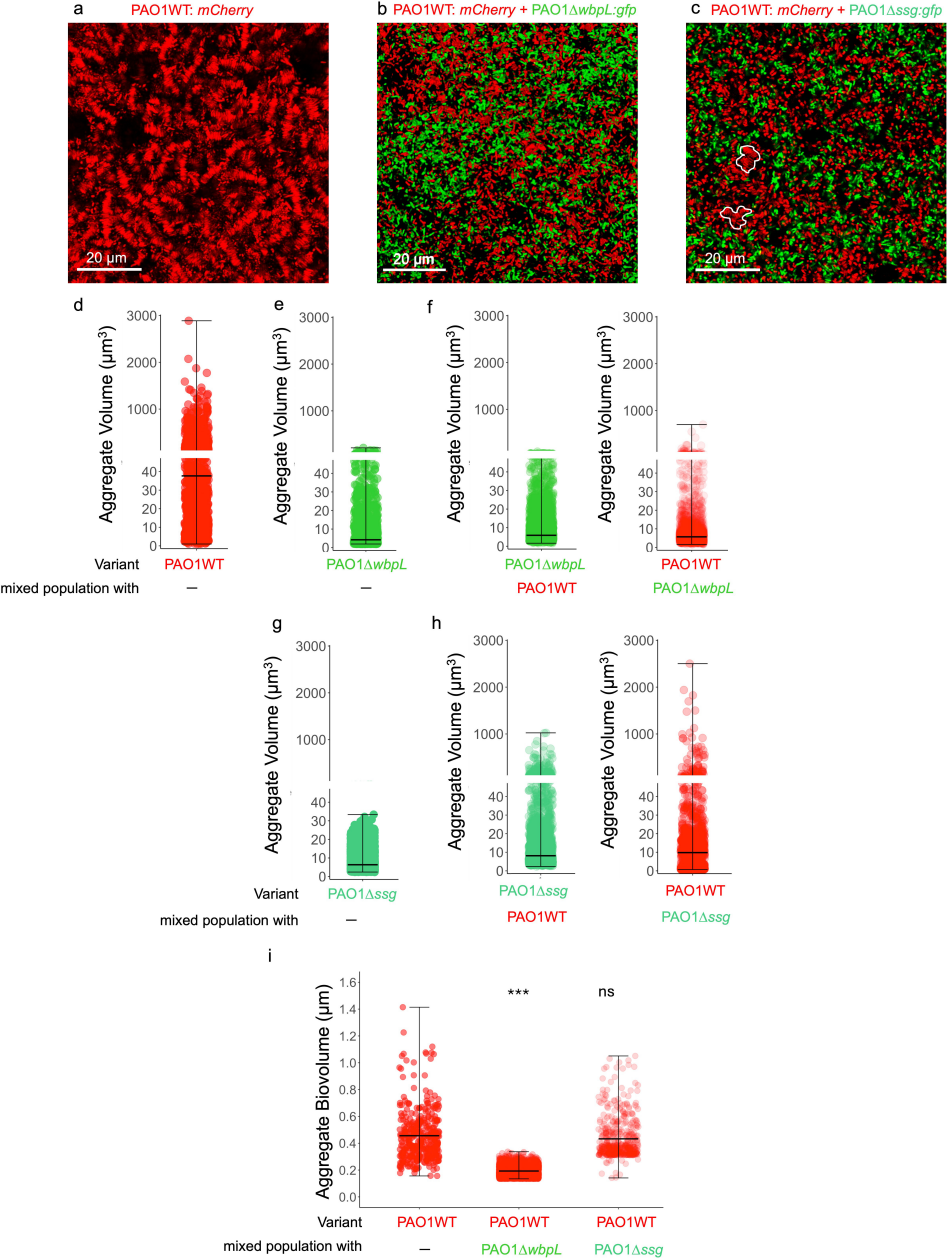
Presence of rough LPS variants alters the biogeography of *P. aeruginosa* populations. (**a**) PAO1WT: *mCherry* cells form stacked aggregates in SCFM2. (**b**) Presence of OSA-deficient variant (PAO1∆*wbpL: gfp*) alters the aggregate assembly of PAO1WT: *mCherry* in mixed populations of PAO1WT + PAO1∆*wbpL*. (**c**) Presence of OSA-deficient variant (PAO1∆*ssg: gfp*) alters the aggregate assembly of PAO1WT: *mCherry* in mixed populations of PAO1WT + PAO1∆*ssg*. (**d**) PAO1WT: *mCherry* cells form large aggregates in SCFM2 (average aggregate volume = 135 µm^3^). (**e**) PAO1∆*wbpL: gfp* cells form small aggregates in SCFM2 (average aggregate volume = 13.3 µm^3^). (**f**) In the presence of PAO1∆*wbpL* cells, PAO1WT cells form significantly smaller aggregates (Kruskal-Wallis chi-squared = 1,388.8, degrees of freedom (df) =1, *Post hoc* pairwise comparisons, Dunn’s multiple comparisons test between PAO1WT and PAO1 +PAO1∆*wbpL; P* < 0.0001). (**g**) PAO1∆*ssg: gfp* cells form small aggregates in SCFM2 (average aggregate volume = 7.63 µm^3^). (**h**) In the presence of PAO1∆*ssg*, PAO1WT cells form smaller aggregates, but the stacked aggregate assembly is not completely disrupted (Kruskal-Wallis chi-squared = 1,388.8, df = 1, *Post hoc* Dunn’s multiple comparisons test between PAO1WT and PAO1 + PAO1∆*ssg; P* = 1.267e-166) (**c**, white outlines) and interestingly, PAO1∆*ssg* cells form significantly larger aggregates when cultured with PAO1WT cells (mixed-population panel, Kruskal-Wallis chi-squared = 351.4, df = 1, *Post hoc* pairwise comparisons, Dunn’s multiple comparisons test between PAO1∆*ssg* and PAO1∆*ssg* + WT, *P* = 3.310e-64). (**i**) There is a significant change in PAO1WT aggregate biovolume in the presence of PAO1∆*wbpL* cells (Welch Twotwo- Ssampleample *t*-test, t = 26.026, df = 360.41, *P*-value < 2.2e-16, ***), indicating disruption of stacked aggregate assembly, while the presence of PAO1∆*ssg* cells does not disrupt stacked aggregate assembly (Welch two-sample *t*-test, df = 676.35, *P*-value = 0.0604, ns). Data presented from three independent experiments, and images are representative of at least 10 independent images acquired from each independent experiment.

To determine whether changes in population structure due to presence of OSA-deficient cells impact the *P. aeruginosa* spatial organization, we first examined the aggregate assembly of mixed *P. aeruginosa* populations *in vitro*. We constructed mixed populations of PAO1 wild-type (PAO1WT) strain with OSA-deficient PAO1∆*wbpL* (PAO1WT + PAO1∆*wbpL*) or PAO1∆*ssg* (PAO1WT + PAO1∆*ssg*) at an initial 1:1 ratio (5 × 10^6^: 5 × 10^6^ CFU) in SCFM2 along with the monoculture parental strain PAO1WT and each OSA-deficient variant SCFM2 in for 24 hours. We then evaluated the changes in spatial organization, total population size, and population structure by comparing the final composition of mixed populations to their initial composition. We found that the presence of PAO1∆*ssg* or PAO1∆*wbpL* disrupts the stacked aggregate assembly of parental PAO1WT strain (Fig. 1a through c; Fig. S1). Interestingly, when grown in a mixed population with OSA-deficient cells, PAO1WT cells formed significantly smaller aggregates in SCFM2 (Fig. 1g and h, and Fig. S1). Although PAO1WT cells formed smaller aggregates in the presence of PAO1∆*ssg* cells, the stacked aggregate assembly was not fully disrupted ( Fig. 1c) as the average of PAO1WT aggregate biovolume (volume/surface area) is higher than 0.2 (15), (biovolume of smallest stacked aggregate of 10 *P*. *aeruginosa* cells = 0.41; PAO1WT_biovolume_ + PAO1∆*ssg* = 0.4327), confirming that stacked aggregate assembly is not fully disrupted in the presence of PAO1∆*ssg,* compared to aggregates formed in the presence of PAO1∆*wbpL* cells (PAO1WT_biovolume_ + PAO1∆*wbpL* = 0.1927) (Fig. 1i).

Interestingly, while the presence of PAO1WT cells did not influence PAO1∆*wbpL* aggregate assembly, it led to assembly of significantly larger aggregates of PAO1∆*ssg* cells (Fig. 1c, h and i ). To assess whether growth in mixed populations influences each variant’s fitness and the population structure, we determined the whole population size by enumerating CFUs and verified the abundance of each variant in mixed populations by qPCR. We found that in mixed populations, the presence of OSA-deficient variants (PAO1∆*ssg* or PAO1∆*wbpL*) significantly increases the population size, suggesting possible cooperative interactions between members of the population (Fig. S2a, Table S1), without influencing the ratio of OSA-deficient cells to PAO1WT compared to the initial 1:1 ratio, or the relative fitness levels of parental PAO1WT strain or either OSA-deficient variants (Fig. S2b and c).

### *P. aeruginosa* forms significantly larger aggregates *in vivo* when co-infected with OSA-deficient variants

We next sought to determine whether the presence of OSA-deficient variants influences the spatial organization of *P. aeruginosa* populations *in vivo*. We employed an established preclinical model for acute airway infection (20, 21) and infected 6-week-old female BALB/c mice (Jackson Laboratories, Bar Harbor, ME) intratracheally with *P. aeruginosa* populations for 24 hours. We focused on determining the role of intrapopulation OSA phenotypic heterogeneity caused by loss of *wbpL* in the murine infection model as WbpL and its homologs play key roles in OSA synthesis and assembly in *P. aeruginosa* and other *Pseudomonas* species (22), while the exact role of Ssg is not clear. Additionally, our *in vitro* studies showed that OSA deficiency due to loss of *wbpL* significantly changes the population spatial organization and aggregate assembly of PAO1WT in SCFM2, while the presence of PAO1∆*ssg* did not fully disrupt the stacked aggregate assembly. Prior to infecting the mice, we cultured PAO1WT, PAO1∆*wbpL*, and a synthetic mixed population of PAO1WT:PAO1∆*wbpL* (1:1 ratio) in SCFM2 for 4–6 hours at 37°C. We added this step prior to infection to ensure that bacterial physiology resembled that observed in CF sputum samples (21, 23). We then infected the mice intratracheally with 25 µL of the standardized inoculum containing 1 × 10^7^ CFU of each bacterial population (five mice for each population). After 24 hours post-infection, we sacrificed the mice to analyze the colonization efficiency and the spatial organization of bacteria in the airways. We quantified the abundance of each *P. aeruginosa* variant by homogenizing the lungs and enumerating CFUs (21), as well as by performing qPCRTable S2. We used the same lung homogenates for genomic DNA isolation and used qPCR to determine the relative abundance of each variant in each lung. Although the change or increase in the size of mixed populations is not clear (Fig. S3a and b), there were no changes in the population structure and relative abundance of PAO1∆*wbpL* cells in airways, either in isolation or as part of mixed populations (Fig. S3c).

**Fig 2:**
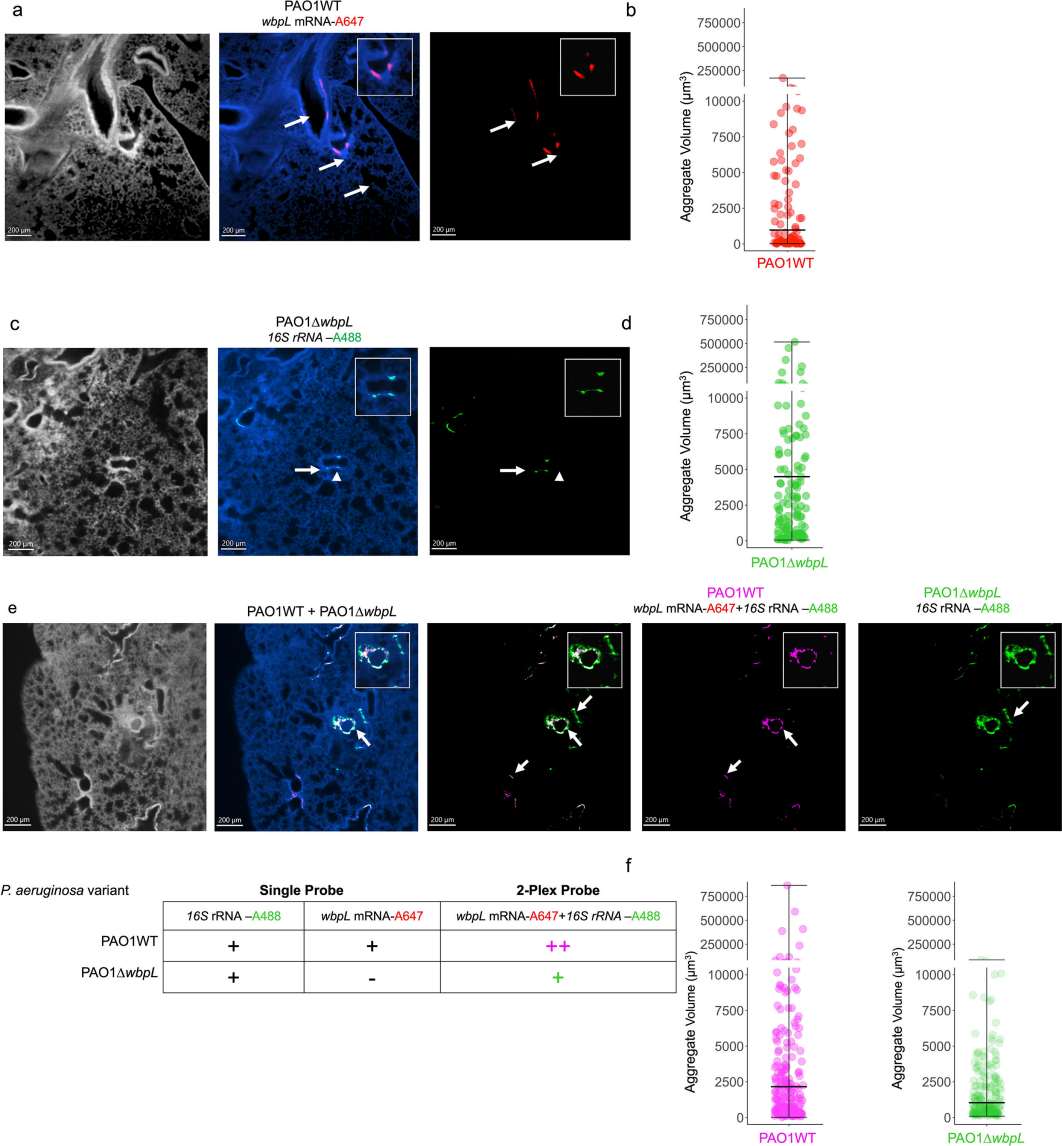
Presence of PAO1∆*wbpL* variants alters airway microbiogeography and increases PAO1WT aggregate size in a murine airway**.** (**a**) PAO1WT cells targeted by the *wbpL-*mRNA-A647 HCR-probe. The aggregates are localized in the central airways. (**b**) The average volume of PAO1 aggregates in murine airways is 7,763 µm^3^. (**c**) PAO1∆*wbpL* cells targeted by the 16S rRNA-A488 HCR-probe. The POA1∆*wbpL* aggregates are mainly localized in smaller airways. (**d**) PAO1∆*wbpL* cells form significantly larger aggregates (average volume = 21,572 µm^3^) in murine airways compared to PAO1WT cells in murine airways (Dunn’s multiple comparisons test *p. adj* = 2.85418328029053e-07). (**e**) Mice infected with mixed populations of PAO1WT + PAO1∆*wbpL*, the PAO1WT cells (in magenta), were tagged with both *wbpL-*mRNA-A647 and 16S rRNA-A488 HCR-probes, whereas the PAO1∆*wbpL* cells are only tagged with the 16S rRNA-A488 HCR probe. (**f**) The PAO1WT cells form significantly larger aggregates when colonizing the lungs in the presence of PAO1∆*wbpL* cells, in comparison to PAO1WT cells in monocultures colonizing the airways (Dunn’s multiple comparisons test, PAO1WT vs PAO1WT + PAO1∆*wbpL*, *p. adj* = 0.0057). Interestingly, the PAO1∆*wbpL* cells formed significantly smaller aggregates in the presence of PAO1WT cells (Dunn’s multiple comparisons test, PAO1∆*wbpL* vs PAO1 +PAO1∆*wbpL*, *p. adj* = 1.01639182104662e-08). In airways colonized with mixed populations, PAO1WT aggregates are also localized in secondary airways along with PAO1∆*wbpL* aggregates (arrows, PAO1WT cells in magenta). Images are representative of at least five images acquired from two infected lungs for each infection condition.

To visualize *P. aeruginosa* cells in the airways, we perfused the lungs with 15 mL of 20 U/mL of heparin in phosphate-buffered saline (PBS). We then fixed the whole lungs in 500 mL of 4% PFA/PBS (vol/vol). We processed the fixed lungs for immunohistochemistry, hybridization chain reaction (HCR) RNA-FISH (24, 25), and tissue-clearing (26) (Fig. S2d). We used HCR RNA-FISH probes targeting *wbpL* mRNA-A647 and 16S rRNA-A488 to identify PAO1WT cells in lungs infected with a mixed population of PAO1WT + PAO1∆*wbpL* (Table S2). We expected to see colocalization of both *wbpL* and 16S rRNA probes only in PAO1WT cells, while cells with only 16S rRNA probes are identified as PAO1∆*wbpL* cells. In mixed populations, we applied the Imaris colocalization pipeline and used automatic thresholding function to detect colocalization of 16S rRNA-A488 (green) and *wbpL* mRNA-A647 (red) HCR probes. We assigned colocalized signals to a new channel representing PAO1WT cells, pseudocolored magenta, and measured their aggregate volumes using the Surface module. Remarkably, the presence of PAO1∆*wbpL* in mixed populations led to a significant increase in PAO1WT aggregate volume from an average of 18,782 µm^3^ in lungs infected only with PAO1WT to an average of 46,701 µm^3^ in lungs infected with mixed populations (Fig. 2d; Movie S1). We also noted that the aggregates detected in the airways are larger than the aggregates formed in SCFM2, suggesting that the presence of OSA-deficient cells may alter cell-cell interactions, resulting in formation of larger aggregates within the airways.

In summary, this study highlights the significant role of *P. aeruginosa* intrastrain population genetic heterogeneity in shaping the microbiogeography of infection, particularly in chronic infections such as CF airways. We found that despite having no measurable effect on the overall population size or each *P. aeruginosa* variant’s relative fitness (Fig. S2b), the presence of PAO1∆*wbpL* significantly alters the spatial organization of *P. aeruginosa* PAO1WT in the mixed populations both *in vitro* and *in vivo*. These findings underscore that intrastrain genetic heterogeneity influences the spatial organization of bacterial populations, potentially modulating microbe-microbe and microbe-host interactions at the micron scale. Such changes in bacterial spatial organizations can facilitate immune evasion and influence the efficacy of antibiotic treatments. Future studies investigating the impacts of intrastrain genetic heterogeneity in clinically sourced *P. aeruginosa* populations on host-pathogen interactions and pathogenesis will be necessary for understanding the broader implications in chronic infections and its impact on airway injury.

## SUPPLEMENTAL MATERIAL

Supplemental Material msphere.00657-25-s0001.pdf Supplemental text, figures, and tables.

Movie S1 msphere.00657-25-s0002.mp4 Aggregate assembly of mixed *P. aeruginosa* populations in murine airways.

ASM does not own the copyrights to Supplemental Material that may be linked to, or accessed through, an article. The authors have granted ASM a non-exclusive, world-wide license to publish the Supplemental Material files. Please contact the corresponding author directly for reuse.
