# Supplementary material for: Whole-tissue imaging reveals intrastrain diversity shapes the spatial organization of Pseudomonas aeruginosa in a murine infection model
Source: mSphere. 2025 Dec 16;11(1):e00657-25. doi: 10.1128/msphere.00657-25 (PMC12838444; doi:10.1128/msphere.00657-25)
Supplement: Supplemental Material — Supplemental text, figures, and tables. [file msphere.00657-25-s0001.pdf]

Whole-Tissue Imaging Reveals Intrastrain Diversity Shapes *Pseudomonas aeruginosa* Spatial Organization in a Murine Infection Model

Fraser H. [a], Moustafa D. A. [b,c], Goldberg J. B. [b,c], Azimi S. [a]

[a]Biology Department, College of Arts and Sciences, Georgia State University, Atlanta, GA, 30303.
[b]Division of Pulmonary, Asthma, Cystic Fibrosis, and Sleep, Department of Pediatrics, Emory University School of Medicine, Atlanta, GA 30322. [c]Emory-Children's Cystic Fibrosis Center, Atlanta, GA 30322.

Supplemental Material

Bacterial strains and growth conditions.

We used the *Pseudomonas aeruginosa* strain PAO1WT and two isogenic mutants, PAO1Δ*ssg* and PAO1Δ*wbpL*, which displays a rough LPS phenotype. We deleted the *wbpL* and *ssg* genes by standard genetic techniques as described previously[1]. Briefly, we introduced 600bp flanking regions of open reading frames of each gene cloned in pEXG2, and introduced this construct to PAO1 parental cells, and selected for cells resistance to 100μg/ml of gentamycin followed by selection of sucrose resistant colonies and gentamicin susceptible clones to ensure the loss of pEXG2 construct. For all *in vitro* experiments in SCFM2[2,3], we first grew the strains in 3 ml of lysogeny broth (LB) at 37˚C/200 *rpm*. We then measured the OD_{600} until they reached mid-log phase. We then measured the OD_{600}, adjusted the cultures to an OD_{600} = 0.01 in 3 ml of SCFM2, and incubated for 4-6 hours for downstream applications such as animal experiments. For imaging we diluted the cultures to an OD_{600} = 0.01 in 400 µl of SCFM2 and incubated them statically at 37˚C. We prepared fresh SCFM2 for each experiment following published protocol[2]. Briefly, we prepared a 50 ml buffered base stock containing basal salts and amino acids, that was adjusted to a pH of 6.8 using hydrochloric acid, and filter sterilized the solution. One day before use, we added 0.6 mg/ml salmon sperm DNA (SIGMA) and 5mg/ml porcine maxillary mucin (SIGMA) to 50 ml of the base. On the day the experiment we supplemented the medium with 500 µl each of dextrose, L-lactic acid, calcium chloride, and magnesium chloride, freshly prepared iron (II)sulphate heptahydrate, N-acetylglucosamine and 1,2- dioleoyl-sn-glycero-3-phosphocholine.

Animal infection model

We grew PAO1 overnight in LB broth for 16-18 h at 37˚C, then suspended the culture in phosphate-buffered saline (PBS) and adjusted OD_{600} to 0.05, corresponding to approximately 10^7 CFU/ml in SCFM2. We incubated the cultures for an additional 6 hours at 37˚C to allow aggregate formation. We adjusted to achieve the desired challenge dose in a volume of 25 µl. We used six-week-old female Balb/c mice (Jackson Laboratories, Bar Harbor, ME) for infection. We infected the mice via non-invasive intratracheal instillation of 50 µL bacterial suspensions, using either single strains (PaO1WT, PAO1Δ*ssg*, or PAO1Δ*wbpL*) or mixed populations. At 24 hours post-infection, we euthanized the mice and either aseptically collected, weighed, and homogenized whole lungs in 1 ml of PBS for serial dilution and plating on Pseudomonas Isolation Agar (PIA) to determine CFU counts[4], or perfused and fixed the lungs in 4% PFA/PBS (w/v) for further tissue clearing. All animal procedures complied with the guidelines of the Emory University Institutional Animal Care and Use Committee, under approved protocol number PROTO201700441.

Quantification of bacterial biomass *in vitro* and *in vivo*

For *in vitro* assays*,* we grew each strain in LB at 37˚C/200 *rpm* to mid-log phase. We then measured the OD_{600} and adjusted the bacterial density to OD_{600} = 0.01 in 3 ml of SCFM2 and incubated statically at 37˚C overnight. We combined PAO1WT and PAO1Δ*ssg* and PAO1Δ*wbpL* at a 1:1 ratio, resulting in a final OD_{600} = 0.01 (OD_{600} = 0.005 for each variant) in 3 ml of SCFM2. To quantify total population size, we used 200 µl from each culture. To disrupt bacterial aggregates, we used 5 mm metal beads and added Tween-20 to cultures (final concentration 1%) and vortexed the samples. We performed 7× 10-fold serial dilutions in PBS and inoculated 5 µl of each dilution onto LB agar plates for CFU enumeration. To process *in vivo* samples, we homogenized the harvested lungs from each mouse in 1 ml of PBS using a bead beater. We then We performed 7× 10-fold serial dilutions in PBS and inoculated 5 µl of each dilution onto LB agar plates for CFU enumeration.

Bacterial abundance analysis using qPCR

We extracted genomic DNA from 1 ml of overnight bacterial cultures in SCFM2 using Wizard Genomic DNA purification kit (Promega), following the manufacturer's instructions. We measured the DNA concentration with a NanoDrop spectrophotometer. For *in vivo* samples, we extracted total DNA from the lung homogenates using the DNeasy Blood and Tissue Kit (Qiagen). For qPCR, we used SYBR Green PCR Master Mix (Applied Biosystems). We diluted all DNA samples to 50 ng/µl and used 250 ng of DNA per reaction. To quantify *P. aeruginosa* abundance, we designed and used three primer sets targeting *gyrA*, *ssg*, and *wbpL* genes for both *in vitro* and *in vivo* experiments (Table. 1).

Hybridization chain reaction fluorescent *in situ* hybridization (HCR-FISH)

We blocked the tissues in 4% (v/v) bovine serum albumin/Tris-Buffered Saline (BSA/TBS) solution for three days at room temperature with gentle shaking. We then permeabilized the whole tissues using Triton X-100. To detect distinct *P. aeruginosa* variants in infected lung tissues, we used specifically designed HCR v3.0 RNA-FISH probes (Molecular Instruments)[5] targeting 16S rRNA and *wbpL* genes in PAO1 (Table 2). Before applying the probes to tissue samples, we validated their specificity in *in vitro* cultures of *P. aeruginosa* by confirming target mRNA binding, following the manufacturer's protocol[5].

Immunofluorescent staining

We diluted the primary antibody, anti-Mouse pan Keratin IgG (Abcam), 1:100 in 4% BSA and incubated the tissues for 1 hour at room temperature. We then washed the tissues once with 0.01% (v/v) of PBS-Tween 20 for 15 minutes, followed by three 15 minutes washes with PBS. We then incubated the tissues overnight at 4°C with the diluted (1:80) secondary antibody, goat anti-rabbit IgG (H&L). We then washed the tissues with PBS-Tween 20 to remove unbound antibodies. We stored the tissues at 4% BSA/TBS solutions at 4°C until further processing.

iDISCO$^+$ tissue clearing

We followed the optimized iDISCO protocol[6]. Briefly, we dehydrated the lung tissues by incubating them in 20%, 40%, 60%, 80%, and 100% methanol in water washes for 1 hour each. We then incubated the samples in a 2:1 mixture of dichloromethane (DCM)/Methanol for 3 hours at room temperature with gentle shaking. Afterward, we washed the tissues twice with 100% DCM for 15 minutes. Finally, we incubated the samples in DiBenzyl Ether (SIGMA) until they became transparent. We stored the cleared tissues in DiBenzyl Ether in dark, at room temperature.

Image acquisition and analysis

We imaged the *in vitro* cultures using an LSM880 confocal laser scanning microscope (Zeiss) as described previously[1]. We imaged whole cleared lung tissues using the MuVi SPIM light-sheet microscope (BRUKER). For each infected lung, we captured up to 700 optical sections in the Z axis with a 50 ms exposure time and a 50 ms delay between frames in the area mode. We used the Surface module in Imaris software (version 10.1.0, Bitplane) to measure aggregate volume and size. For each lung, we analyzed three independent image fields to quantify bacterial aggregates' volume and size. For *in vitro* cultures, we quantified aggregate volumes of at least 10 independently acquired images across three independent biological replicates per condition.

Data visualization and statistical analysis

We used R v4.5.0 for all statistical analyses and data visualization.

Fig. S1.

a

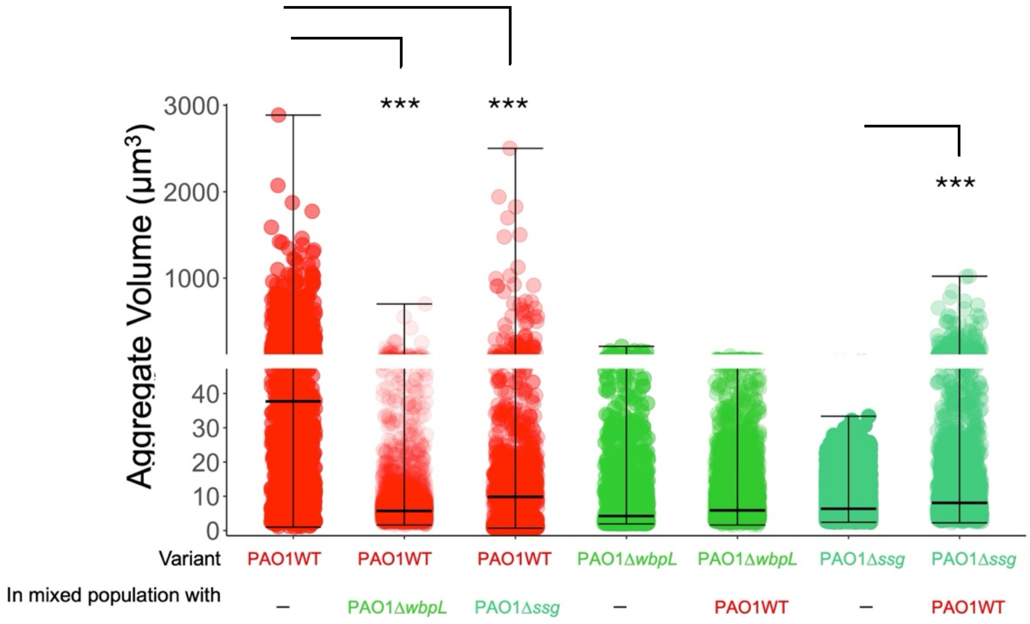

b

Aggregate Volume (μm)	Minimum (μm)	Maximum (μm)	Median (μm)
PAO1WT	1	2887	37.7
PAO1WT in presence of PAO1Δ*wbpL*	1.62	701	5.70
PAO1WT in presence of PAO1Δ*ssg*	0.701	2501	9.81
PAO1Δ*wbpL*	1.95	210	4.23
PAO1Δ in presence of PAO1WT	1.62	98.t	5.89
PAO1Δ*ssg*	2.42	33.3	6.36
PAO1Δ*ssg* in presence of PAO1WT	2.27	1022	8.06

c

Group1	Group2	*p*	*p.adj*	*p.adj.signif*
PAO1Δ*ssg*	PAO1Δ*ssg* in presence of PAO1WT	3.310e-64	1.65e-63	****
PAO1WT	PAO1WT in presence of PAO1Δ*ssg*	1.267e-166	1.01e-165	****
PAO1WT	PAO1WT in presence of PAO1Δ*wbpL*	<0.0001	<0.0001	****

Fig S1. Presence of OSA deficient variants significantly alters aggregate assembly by PAO1WT cells. (a) Presence of OSA-deficient variants PAO1Δ*wbpL: gfp* and PAO1Δ*ssg: gfp* lead to formation of significantly smaller aggregates of PAO1WT: *mCherry* cells in mixed populations (b) The distribution of *P. aeruginosa* aggregate volume in different growth conditions tested here. (c) Results of Post-hoc Dunn's multiple comparisons test.

Fig. S2.

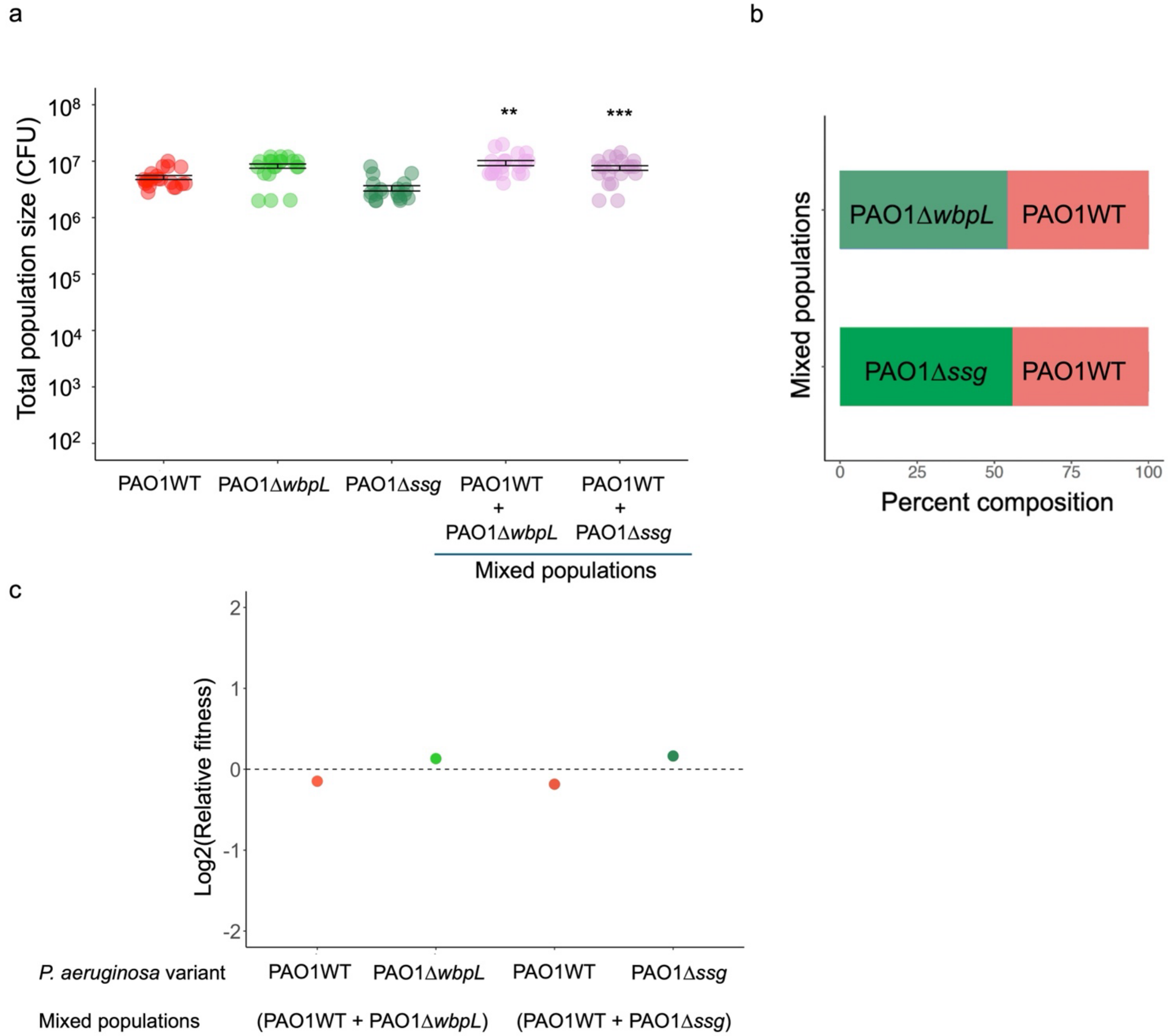

Fig. S2. Presence of OSA deficient variants increases total population size *in vitro*. (a) Mixed populations of PAO1WT+PAO1Δ*ssg* and PAO1WT+PAO1Δ*wbpL* grow to higher population size compared to PAO1 in isolation (data presented from five independent experiments, One-way ANOVA, (F (4, 105) = 13.22, Post-hoc pairwise comparisons, Tukey's multiple comparisons test between PAO1WT, PAO1WT+PAO1Δ*ssg* ; p=0.015, and PAO1WT+PAO1Δ*wbpL*; p= 0.00043). (b) Despite an increase in the mixed population size, the population structures remained the same as initial 1:1 ratio of OSA-deficient: PAO1 cells, after 24 hours of growth in SCFM2. (c) We calculated the relative fitness of each variant based on the changes in initial and final abundance in mixed populations. There are no significant changes in relative fitness of each variant in mixed populations.

Figure S3.

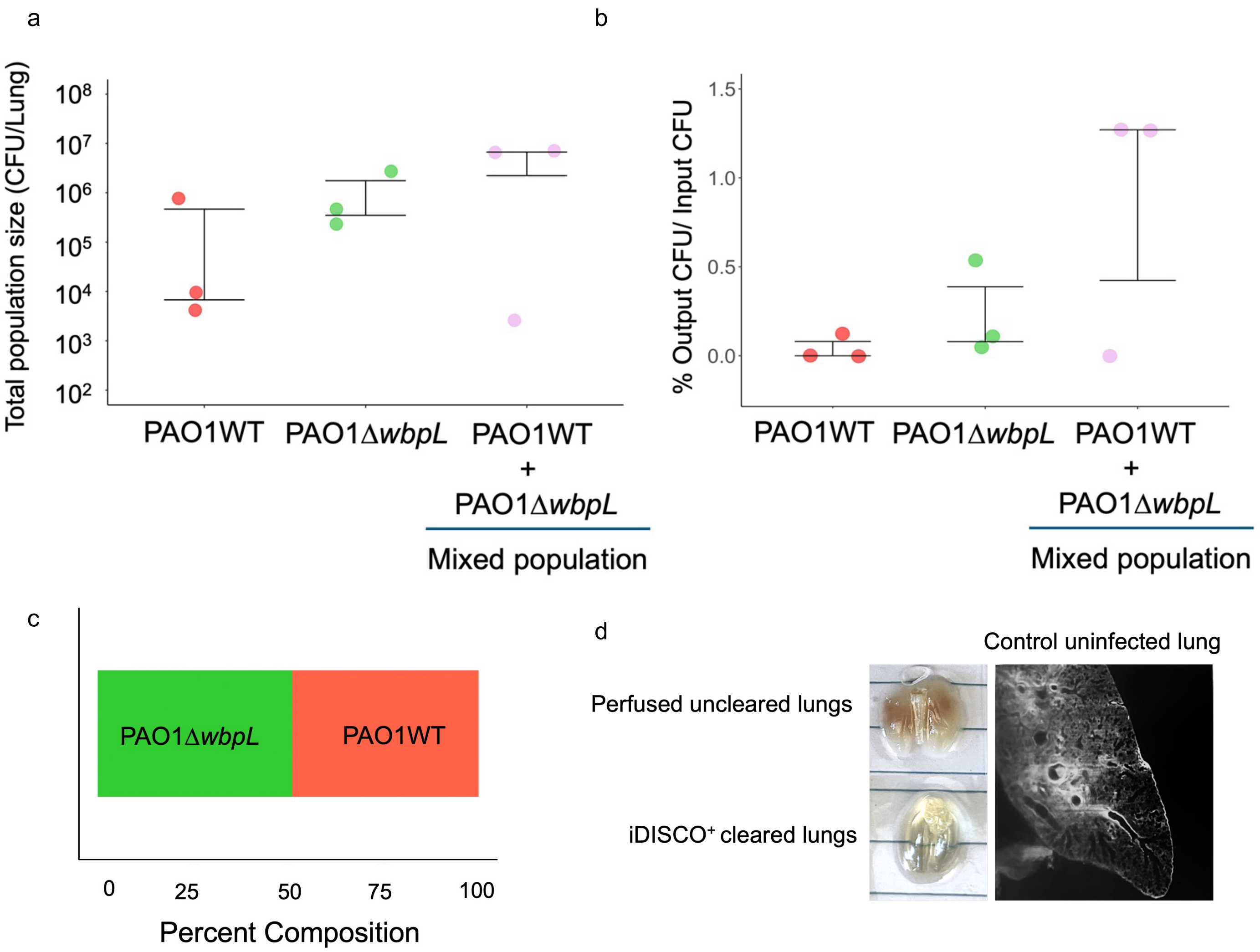

Fig. S3. Presence of OSA deficient PAO1Δ*wbpL* does not change total bacterial load in mouse lungs. (a) There are no significant changes in *P. aeruginosa* population size in mice infected with mixed population of PAO1WT+PAO1Δ*wbpL* (Kruskal-Wallis, df = 1, $p = 0.5066$). (b) The difference in colonization rate of different *P. aeruginosa* populations was not statistically significant, possibly due to the low number of samples and high variability among samples (Data presented from three mice individually infected with single variant or mixed cultures). (c) *P. aeruginosa* population structure remained the same as initial 1:1 ratio of PAO1Δ*wbpL*: PAO1WT in lungs 24 hours post infection. (d) Use of iDISCO+ tissue clearing allows for whole lung imaging.

Table S1. Primer sets used in this study.

ssg 1	TTTTCCCGCTCGCCCTGGTCGT
ssg 2	TGCTCGACGAGTTGGGCGGAGT
wbpL 1	GGCGTGGCACTGTTAGGGTTCC
wbpL 2	TTCCAGATCAGGAAGCCGGCGA
gyrA 1	TGTGCTTTATGCCATGAGACGA
gyrA 2	CGTTGAAGCCAAGCCACCT

Table S2. HCR Probe-binding Sequences.

Name	Probe-binding Sequences
wbpL 1	TTTTCCAAGGAACCCGCCTTGTATCCTACCGCCAAGAGGCAAAGA
wbpL 2	AGCATAAGCAACCAGGATACCGATGAAGCCGCTGATCCAACCCAATGCAACC
wbpL 3	CAGTGCCAACGGCAACAACCAAAGAGTGTTGATAGCCAGAACACCCAAGGTA
wbpL 4	CCGCAGATGGCTAGCATAACGACGCGAGGCAAACTGATAAGCGTGGCTGCGA
wbpL 5	CGCCTCATAGAATTTCTCCCCTCTGGCGATCCGGCGGATCAGAGTATAGGTT
wbpL 6	GGTCCATGCAGCCTGAATAGCTAGTGCACCAATAACCATACCAAGAAAACCA
wbpL 7	CCCCGCATCACCCATGAAGATTCGAGCTGGAGGGAAGTTCCAGATCAGGAAG
wbpL 8	GGCGACCGCGCACGCCAGCAACAGAGGGATACCAACCATCGCGACATGCCCT
wbpL 9	AAGCCAGTAGATCAGGGCCCCTCCTACACAGACACCAATGGCCTCGACACTG
wbpL 10	AATACCATCAATGCCATCCATGAAGTTATAAAGGTTCAGCACCCATACCAAA
wbpL 11	GAAAACTGCCAATACGTGGCCCAGCCATCCTAAGTCGACAGCATGCCCAACC
wbpL 12	ATGGCCGAGCAGCCGCCAACGCGCAGCAATGTGCCCATGGTCATCCAGGAAC
wbpL 13	GATACTGCCTGCACTCAGCATCCACACCAACGCTGCAAGGAAGACCAGAACG
wbpL 14	TGCAACACCTCCCCCCCTAGGCGTCGGTTGACTGTGGGAGCTACGGGCATTC
wbpL 15	AACATCCATCAGTTTCGTCGCTAATGCATAGCGACGCAGCCCCCAGGTAGCG
16S rRNA 1	GAACTGAAGAGTTTGATCATGGCTCAGATTGAACGCTGGCGGCAGGCCTAAC
16S rRNA 2	CAAAGGGTTGCCAAGCCGCGAGGTGGAGCTAATCCCATAAAACCGATCGTAG
16S rRNA 3	CAAGTCATCATGGCCCTTACGGCCAGGGCTACACACGTGCTACAATGGTCGG
16S rRNA 4	GGGTTAAGTCCCGTAACGAGCGCAACCCTTGTCCTTAGTTACCAGCACCTCG
16S rRNA 5	GGAACTCAGACACAGGTGCTGCATGGCTGTCGTCAGCTCGTGTCGTGAGATG
16S rRNA 6	CCTTACCTGGCCTTGACATGCTGAGAACTTTCCAGAGATGGATTGGTGCCTT
16S rRNA 7	TAAGTCGACCGCCTGGGGAGTACGGCCGCAAGGTTAAAACTCAAATGAATTG
16S rRNA 8	CGATGTCGACTAGCCGTTGGGATCCTTGAGATCTTAGTGGCGCAGCTAACGC
16S rRNA 9	GCGAAAGCGTGGGGAGCAAACAGGATTAGATACCCTGGTAGTCCACGCCGTA
16S rRNA 10	GGAAGGAACACCAGTGGCGAAGGCGACCACCTGGACTGATACTGACACTGAG
16S rRNA 11	GATGTGAAATCCCCGGGCTCAACCTGGGAACTGCATCCAAAACTACTGAGCT
16S rRNA 12	AGCGTTAATCGGAATTACTGGGCGTAAAGCGCGCGTAGGTGGTTCAGCAAGT
16S rRNA 13	AGAATAAGCACCGGCTAACTTCGTGCCAGCAGCCGCGGTAATACGAAGGGTG
16S rRNA 14	TAAGTTGGGAGGAAGGGCAGTAAGTTAATACCTTGCTGTTTTGACGTTACCA
16S rRNA 15	CGACGATCCGTAACTGGTCTGAGAGGATGATCAGTCACACTGGAACTGAGAC
16S rRNA 16	ATCAGATGAGCCTAGGTCGGATTAGCTAGTTGGTGGGGTAAAGGCCTACCAA
16S rRNA 17	CTAATACCGCATACGTCCTGAGGGAGAAAGTGGGGGATCTTCGGACCTCACG
16S rRNA 18	ATGCAAGTCGAGCGGATGAAGGGAGCTTGCTCCTGGATTCAGCGGCGGACGG
16S rRNA 19	CGGATCGCAGTCTGCAACTCGACTGCGTGAAGTCGGAATCGCTAGTAATCGT
16S rRNA 20	ACCACGGAGTGATTCATGACTGGGGTGAAGTCGTAACAAGGTAGCCGTAGGG

References

1. Azimi, S. *et al.* O-Specific Antigen-Dependent Surface Hydrophobicity Mediates Aggregate Assembly Type in *Pseudomonas aeruginosa*. *mBio*, e0086021 (2021). https://doi.org/10.1128/mBio.00860-21
2. Turner, K. H., Wessel, A. K., Palmer, G. C., Murray, J. L. & Whiteley, M. Essential genome of *Pseudomonas aeruginosa* in cystic fibrosis sputum. *Proc Natl Acad Sci U S A* **112**, 4110-4115 (2015). https://doi.org/10.1073/pnas.1419677112
3. Cornforth, D. M. *et al. Pseudomonas aeruginosa* transcriptome during human infection. *P Natl Acad Sci USA* **115**, E5125-E5134 (2018). https://doi.org/10.1073/pnas.1717525115
4. Mukherjee, S. *et al.* The PqsE and RhlR proteins are an autoinducer synthase-receptor pair that control virulence and biofilm development in *Pseudomonas aeruginosa*. *Proc Natl Acad Sci U S A* **115**, E9411-E9418 (2018). https://doi.org/10.1073/pnas.1814023115
5. Choi, H. M. T. *et al.* Third-generation in situ hybridization chain reaction: multiplexed, quantitative, sensitive, versatile, robust. *Development* **145** (2018). https://doi.org/10.1242/dev.165753
6. Habart, M., Lio, G., Soumier, A., Demily, C. & Sirigu, A. An optimized iDISCO+ protocol for tissue clearing and 3D analysis of oxytocin and vasopressin cell network in the developing mouse brain. *STAR Protoc* **4**, 101968 (2023). https://doi.org/10.1016/j.xpro.2022.101968
